# Supplementary material for: The N-terminus of GPR37L1 is proteolytically processed by matrix metalloproteases
Source: Sci Rep. 2020 Nov 17;10:19995. doi: 10.1038/s41598-020-76384-9 (PMC7673139; doi:10.1038/s41598-020-76384-9)

The N-terminus of GPR37L1 is proteolytically processed by matrix metalloproteases

James L. J. Coleman†[1,2], Tony Ngo†[1,2], Rhyll E. Smythe[1,3], Andrew J. Cleave[1,3], Nicole M. Jones[3], Robert M. Graham[1,2], Nicola J. Smith*[1,2,3,4]

Affiliations

[1]Molecular Cardiology & Biophysics Division, Victor Chang Cardiac Research Institute, Darlinghurst, Australia.

[2]St Vincent's Clinical School, UNSW Sydney, Darlinghurst, Australia.

[3]Department of Pharmacology, School of Medical Sciences, UNSW Sydney, Kensington, Australia.

[4]Orphan Receptor Laboratory, School of Medical Sciences, UNSW Sydney, Kensington, Australia

*To whom correspondence should be addressed: Dr Nicola J Smith, nicola.smith@unsw.edu.au

†These authors contributed equally to this work.

Supplementary Figure

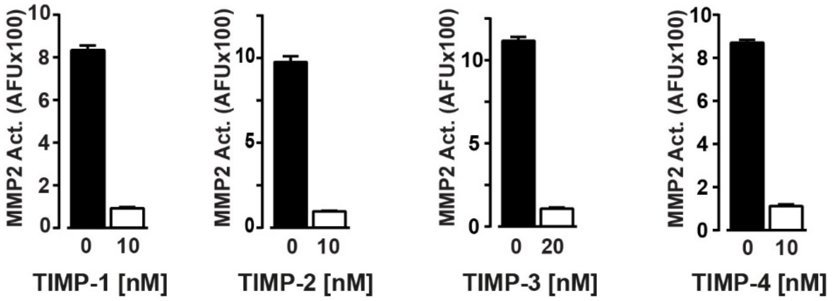

Supp 1: Human recombinant Tissue Inhibitors of Metalloproteases were biologically active.

Human recombinant TIMP-1, TIMP-2, TIMP-3, or TIMP-4 biological activity was verified *in vitro* using activated MMP2 and the fluorogenic substrate, MCA-Pro-Leu-Gly-Leu-DPA-Ala-Arg-NH_2, as per the protocol provided by R&D Systems. AFU: arbitrary fluorescence units.

Figure 1 GPR37L1 exists as both a full-length and N-terminally truncated species.

Figure 1: Coleman, Ngo et al. *Scientific Reports*

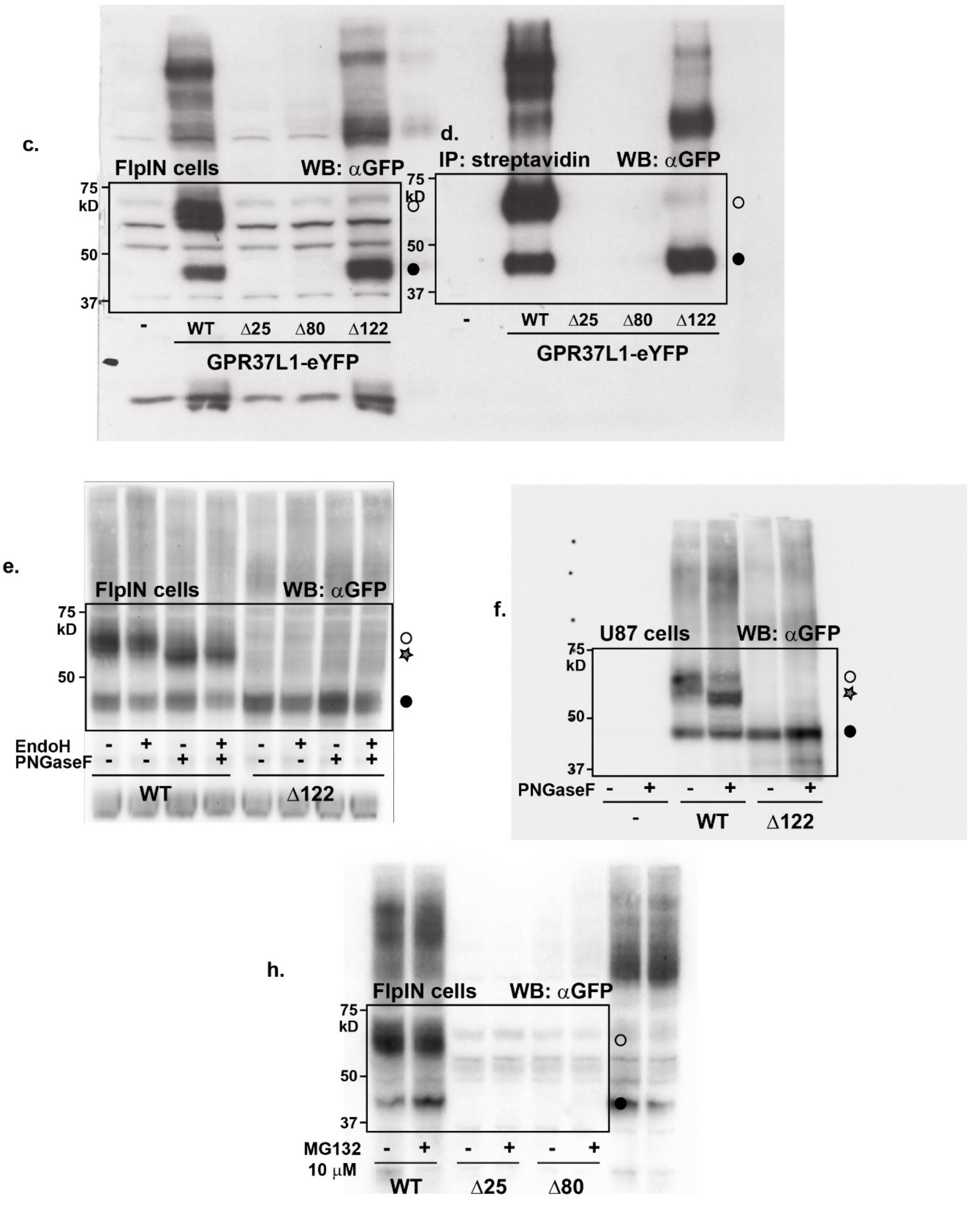

Figure 2 GPR37L1 is processed at the N-terminus by an ADAM.

Figure 2a-c: Coleman, Ngo et al. *Scientific Reports*

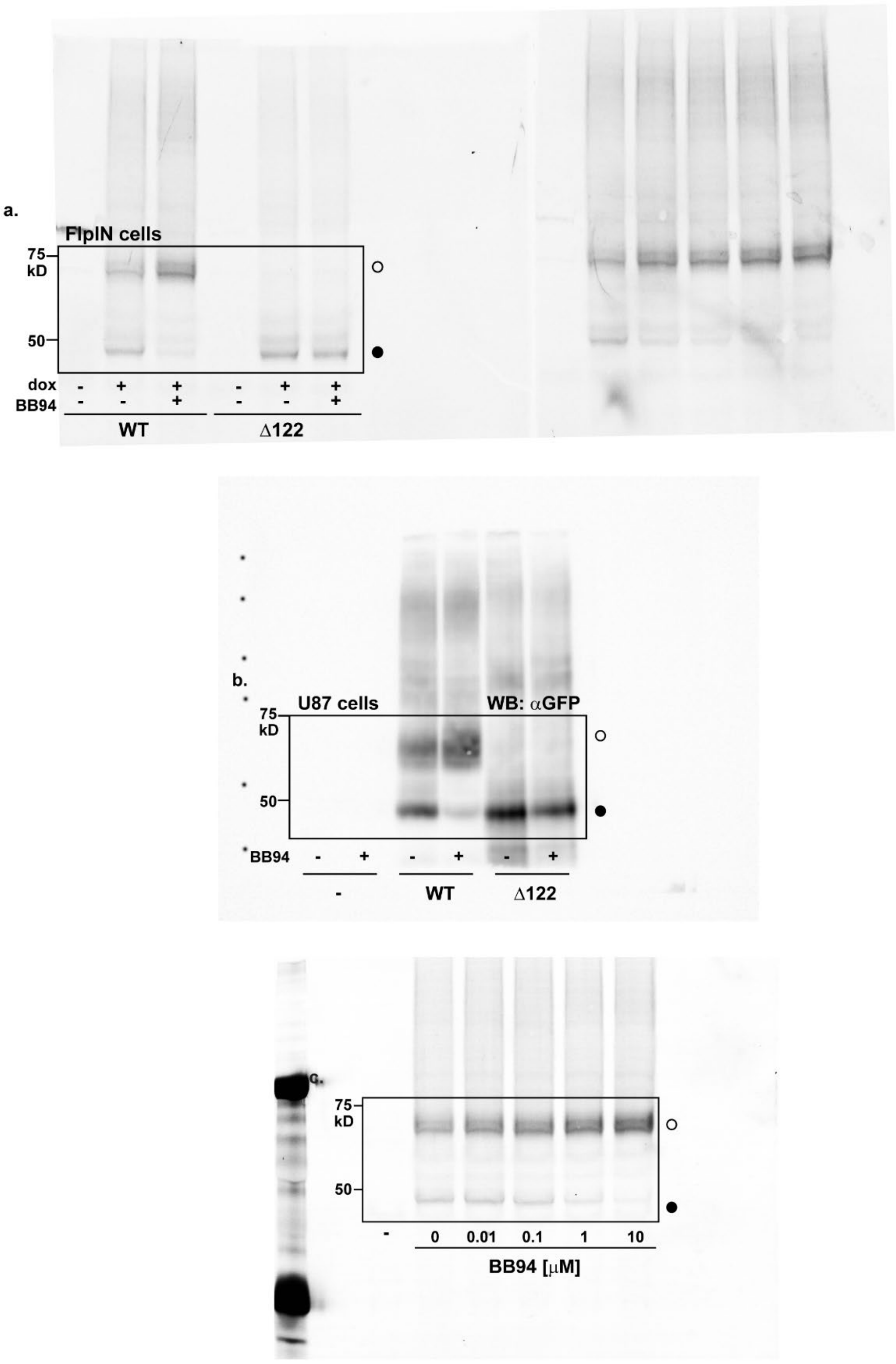

Figure 2d: Coleman, Ngo et al. *Scientific Reports*

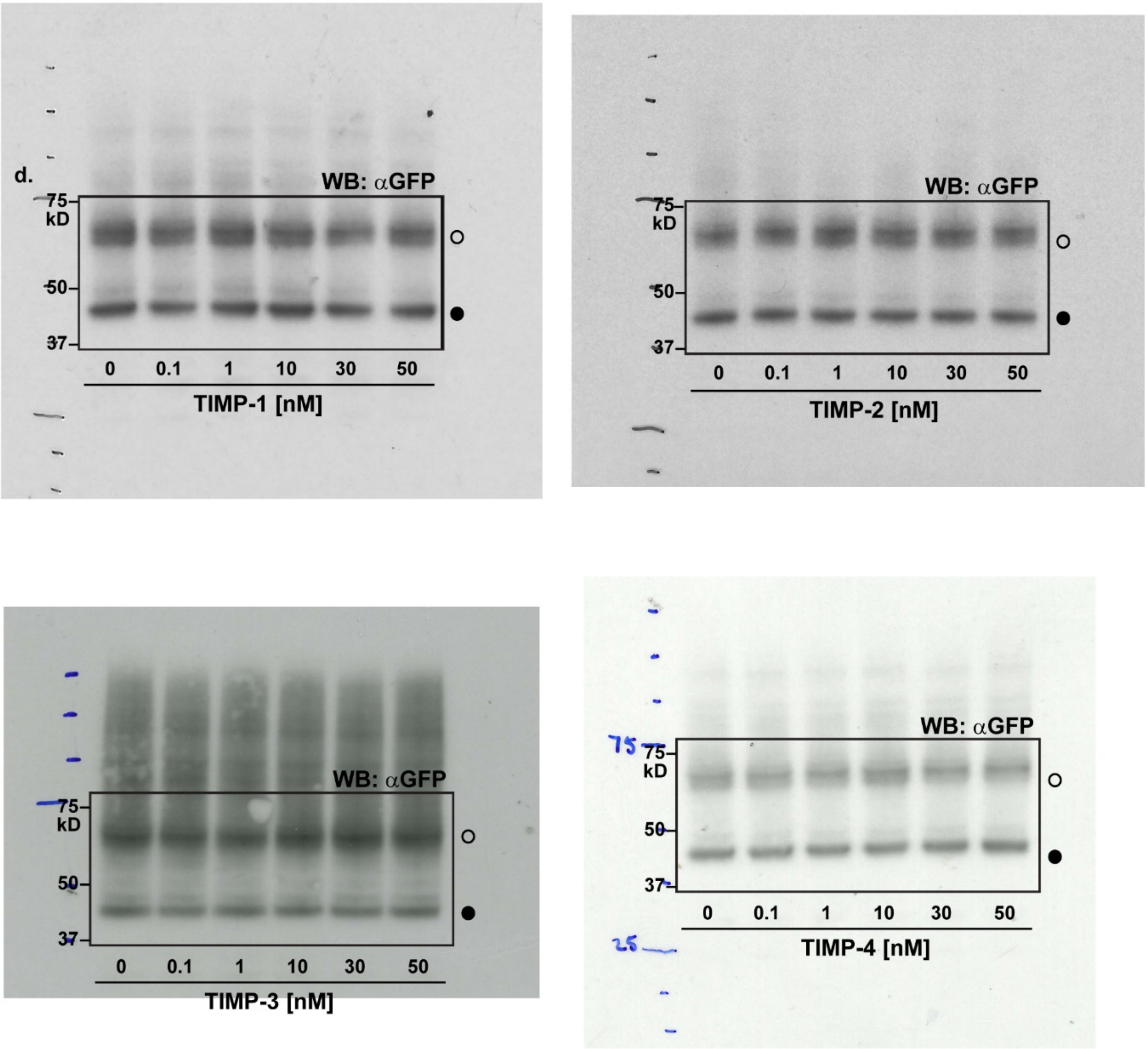

Figure 2e-f: Coleman, Ngo et al. *Scientific Reports*

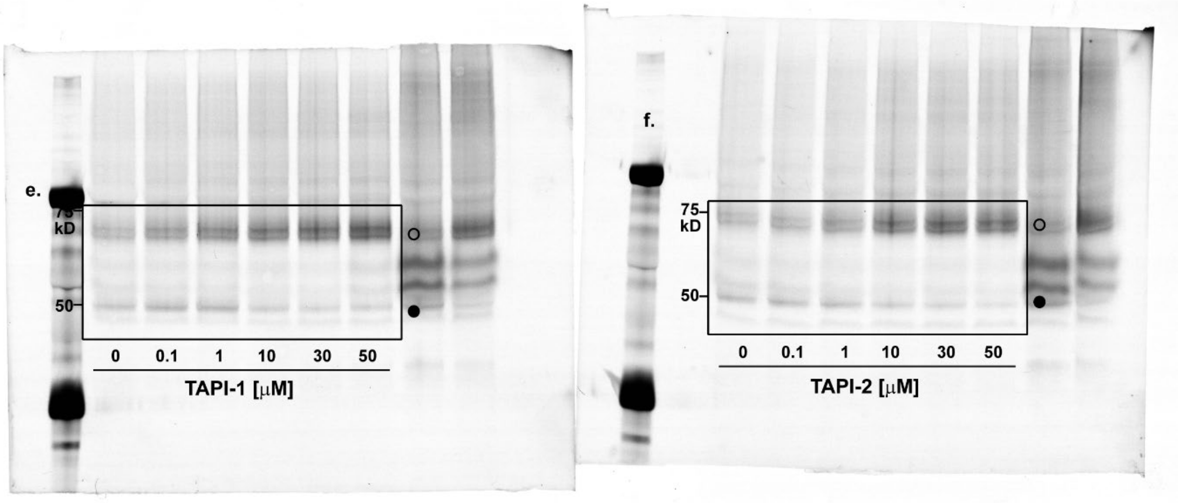

Figure 3 Systematic triplet alanine scanning mutation reveals residues necessary for GPR37L1-eYFP glycosylation and expression.

Figure 3b: Coleman, Ngo et al. *Scientific Reports*

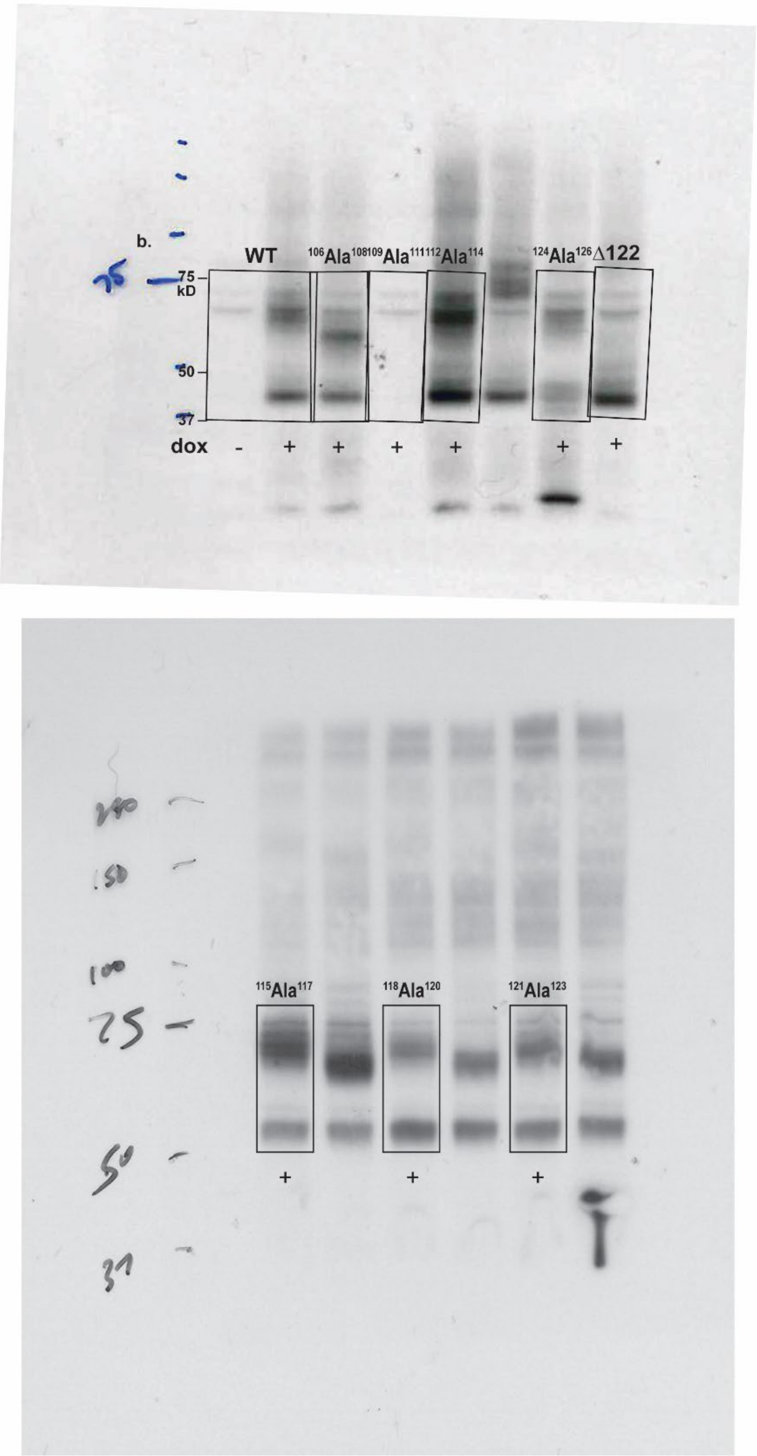

Figure 3c: Coleman, Ngo et al. *Scientific Reports*

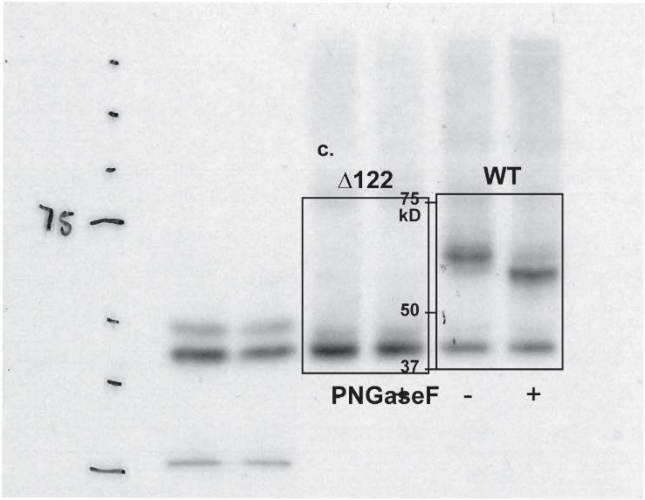

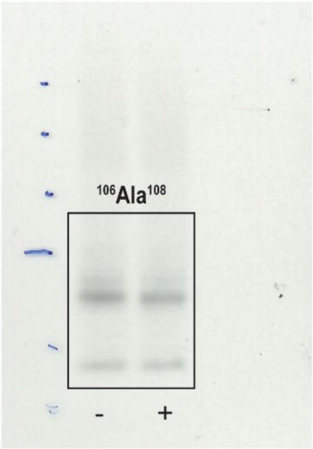

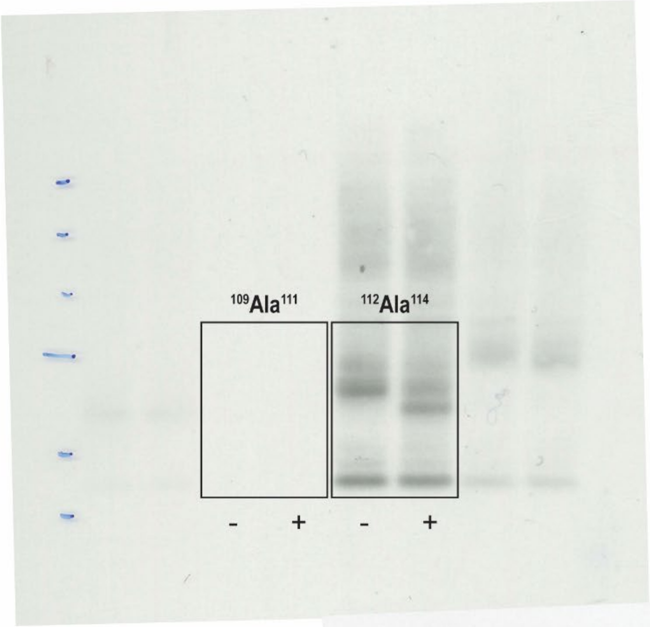

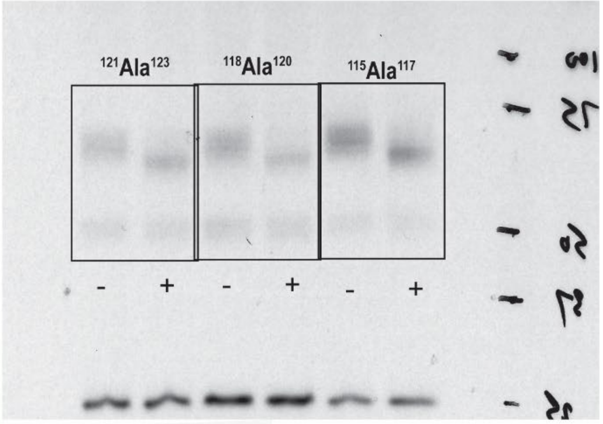

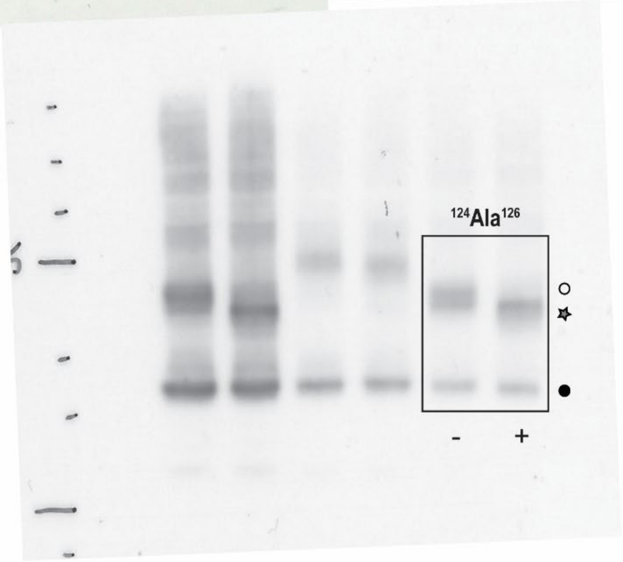

Figure 3d: Coleman, Ngo et al. *Scientific Reports*

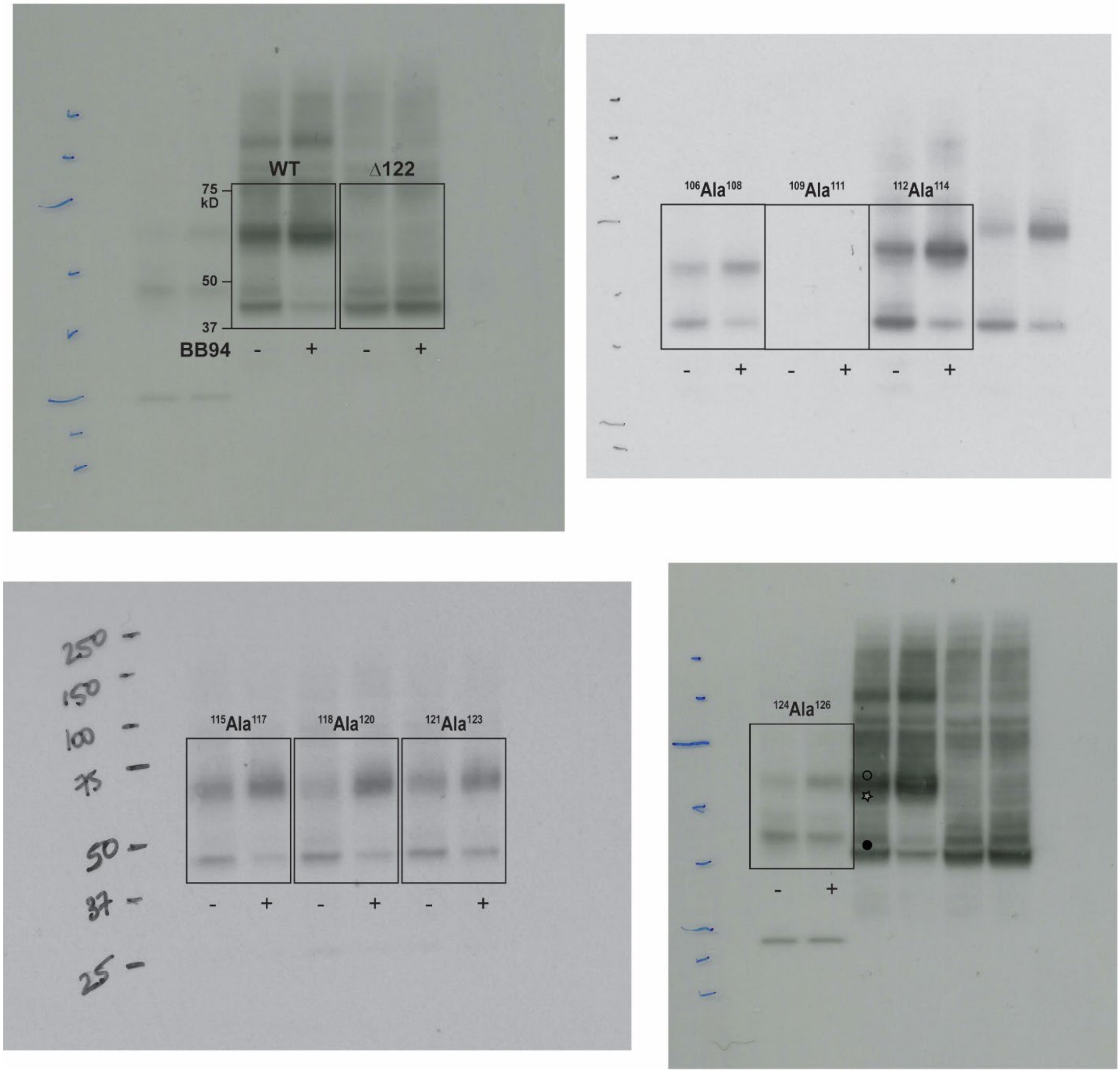

Figure 5 GPR37L1 is N-terminally proteolytically processed in vivo.

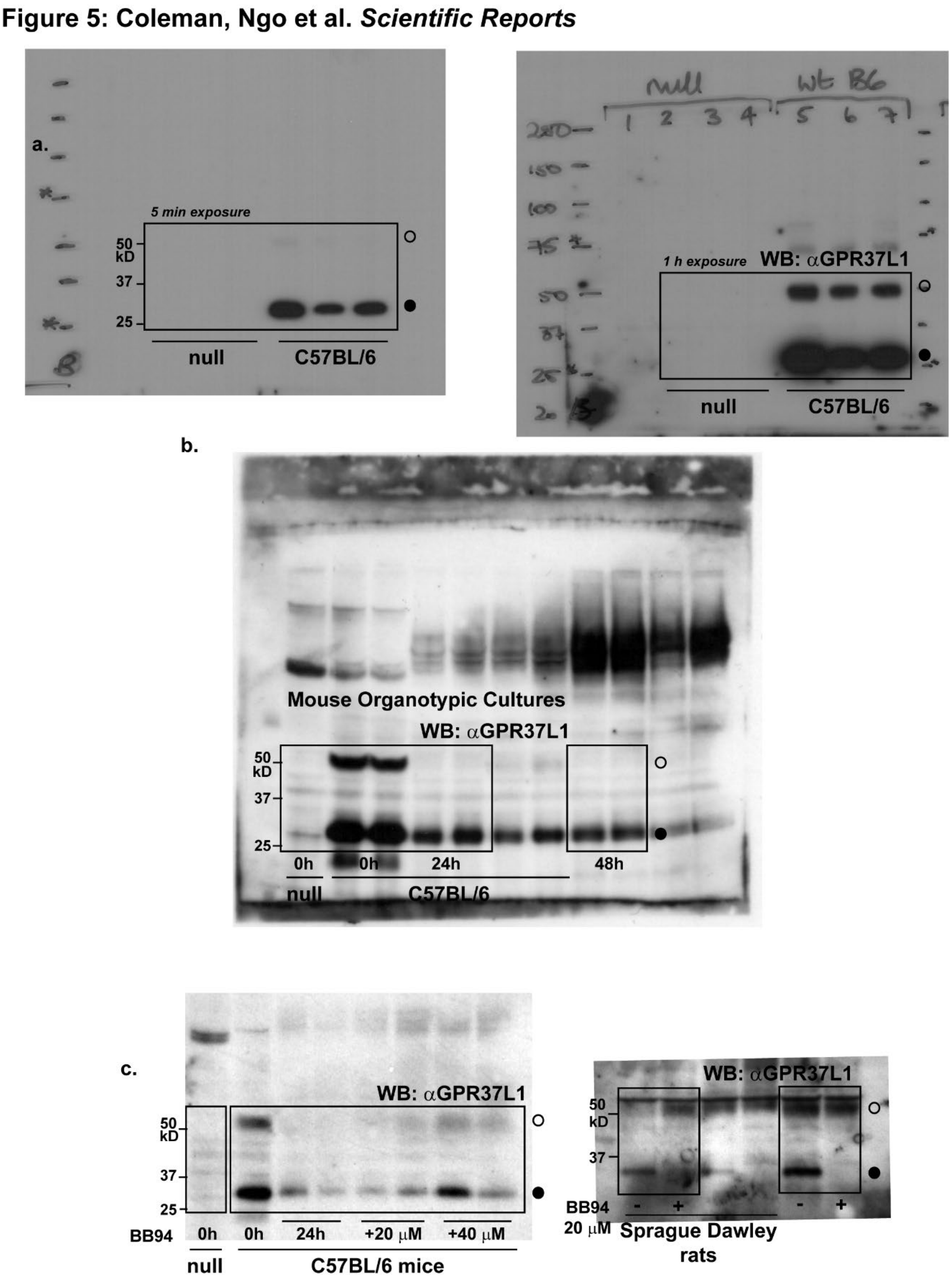

Figure 6 TX14A does not stimulate GPR37L1 signaling or alter protein abundance

Figure 6: Coleman, Ngo et al. *Scientific Reports*

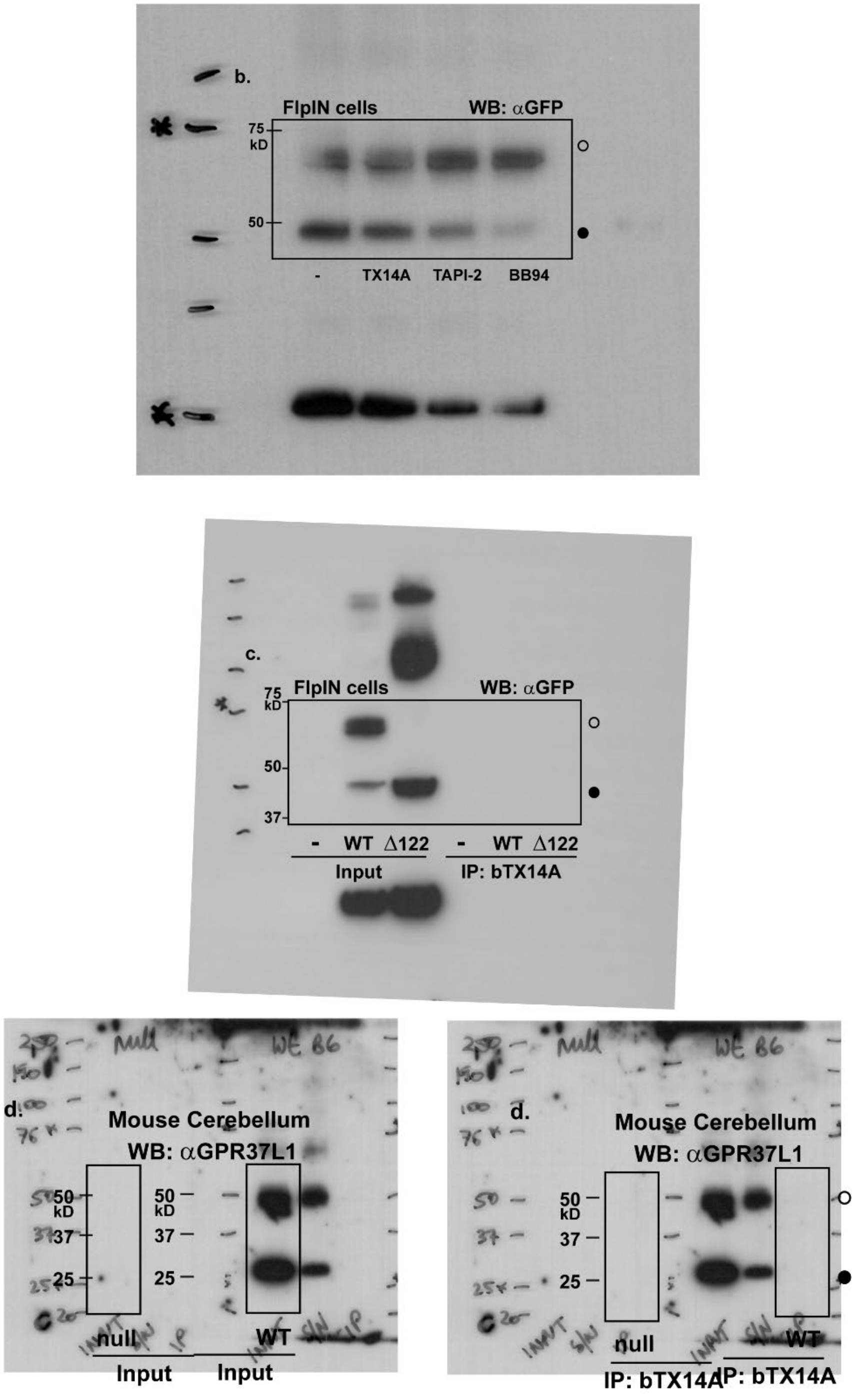

Supplement: Supplementary file 1 — Supplementary Information. [file 41598_2020_76384_MOESM1_ESM.pdf]
